# A nomogramic model based on clinical and laboratory parameters at admission for predicting the survival of COVID-19 patients

**DOI:** 10.1186/s12879-020-05614-2

**Published:** 2020-11-30

**Authors:** Xiaojun Ma, Huifang Wang, Junwei Huang, Yan Geng, Shuqi Jiang, Qiuping Zhou, Xuan Chen, Hongping Hu, Weifeng Li, Chengbin Zhou, Xinglin Gao, Na Peng, Yiyu Deng

**Affiliations:** 1 Department of Infectious Diseases, Guangdong Provincial People’s Hospital, Guangdong Academy of Medical Sciences, Guangzhou, 510080 Guangdong Province China; 2 Department of Critical Care Medicine, Guangdong Provincial People’s Hospital, Guangdong Academy of Medical Sciences, 106 Zhongshan 2nd Road, Guangzhou, 510080 Guangdong China; 3 Departments of Respiratory and Critical Care Medicine, Guangdong Provincial People’s Hospital, Guangdong Academy of Medical Sciences, Guangdong Zhongshan 2nd Road NO.106, Guangzhou, 510080 Guangdong China; 4 Department of Digestive, NO. 923 Hospital of Joint Service Supporting Force, Nanning, 530021 Guangxi China; 5 School of Medicine, South China University of Technology, 381 Wushan Road, Tianhe District, Guangzhou, 510006 Guangdong China; 6 Shantou University Medical College, 243 Daxue Road, Shantou, 5105063 Guangdong China; 7 Department of Emergency, Wuhan Hankou Hospital, 2273 Jiefang Avenue, Wuhan, 430010 Hubei China; 8 Department of Emergency and Critical Care Medicine, Guangdong Provincial People’s Hospital, Guangdong Academy of Medical Sciences, Guangzhou, China; 9 Department of Cardiovascular Surgery, Guangdong Cardiovascular Institute, Guangdong Provincial Key laboratory of South China Structural Heart Disease, Guangdong Provincial People’s Hospital, Guangdong Academy of Medical Sciences, 106 Zhongshan 2nd Road, Guangzhou, 510080 Guangdong China; 10 Department of Critical Care Medicine, General Hospital of Southern Theater Command of PLA, Guangzhou, 510010 Guangdong China; 11 China Department of Critical Care Medicine, Huo Shenshan Hospital of Wuhan, Wuhan, 430199 Hubei China

**Keywords:** SARS-CoV-2, COVID-19, Risk factor, Prediction model, Nomogram

## Abstract

**Background:**

COVID-19 has become a major global threat. The present study aimed to develop a nomogram model to predict the survival of COVID-19 patients based on their clinical and laboratory data at admission.

**Methods:**

COVID-19 patients who were admitted at Hankou Hospital and Huoshenshan Hospital in Wuhan, China from January 12, 2020 to March 20, 2020, whose outcome during the hospitalization was known, were retrospectively reviewed. The categorical variables were compared using Pearson’s χ^2^-test or Fisher’s exact test, and continuous variables were analyzed using Student’s *t*-test or Mann Whitney *U*-test, as appropriate. Then, variables with a *P-*value of ≤0.1 were included in the log-binomial model, and merely these independent risk factors were used to establish the nomogram model. The discrimination of the nomogram was evaluated using the area under the receiver operating characteristic curve (AUC), and internally verified using the Bootstrap method.

**Results:**

A total of 262 patients (134 surviving and 128 non-surviving patients) were included in the analysis. Seven variables, which included age (relative risk [RR]: 0.905, 95% confidence interval [CI]: 0.868–0.944; *P* < 0.001), chronic heart disease (CHD, RR: 0.045, 95% CI: 0.0097–0.205; *P* < 0.001, the percentage of lymphocytes (Lym%, RR: 1.125, 95% CI: 1.041–1.216; *P* = 0.0029), platelets (RR: 1.008, 95% CI: 1.003–1.012; *P* = 0.001), C-reaction protein (RR: 0.982, 95% CI: 0.973–0.991; *P* < 0.001), lactate dehydrogenase (LDH, RR: 0.993, 95% CI: 0.990–0.997; *P* < 0.001) and D-dimer (RR: 0.734, 95% CI: 0.617–0.879; *P* < 0.001), were identified as the independent risk factors. The nomogram model based on these factors exhibited a good discrimination, with an AUC of 0.948 (95% CI: 0.923–0.973).

**Conclusions:**

A nomogram based on age, CHD, Lym%, platelets, C-reaction protein, LDH and D-dimer was established to accurately predict the prognosis of COVID-19 patients. This can be used as an alerting tool for clinicians to take early intervention measures, when necessary.

## Background

Coronavirus disease 2019 (COVID-19), which is caused by severe acute respiratory syndrome coronavirus 2 (SARS-CoV-2), has become a pandemic and major threat to global health [1–4]. As of June 2, 2020, more than 6.2 million cases have been reported worldwide, with a death toll approaching 370,000 (https://www.who.int/docs/default-source/coronaviruse/situation-reports). In clinic, 10–15% of patients with COVID-19 develop severe or critical outcomes, such as acute respiratory distress syndrome (ARDS), and eventually, multiple organ dysfunction or even death [5]. At present, there are no specific drugs for SARS-CoV-2, and the treatment for COVID-19 depends on the severity of the manifestations developed during the disease course. Theoretically, the early prediction of the subsequent development of severe or critical outcomes, including death, with early intervention would help greatly prevent severe, critical, or fatal consequences. Therefore, there is an urgent need to establish and validate models to accurately predict patient outcomes, in order to implement early intervention, and thereby optimize patient care and resource allocation during the pandemic.

A nomogram is known to be a commonly used visual tool that integrates clinical data and laboratory variables for predicting the outcome of a disease. It was invented in the nineteenth century and has been widely used as a visualized, intuitive and appreciable tool in clinical practice [6–8]. Therefore, the present study aimed to develop a predictive nomogram model to predict the survival of COVID-19 patients based on their baseline clinical and laboratory data at admission.

## Methods

### Patients

Patients who were diagnosed with COVID-19, and admitted at Hankou Hospital and Huoshenshan Hospital in Wuhan, China from January 12, 2020 to March 20, 2020, whose outcome (survived [recovered and discharged from the hospital] or did not survive [died] during the hospitalization) was known by April 24, 2020, were retrospectively reviewed. Their demographic, epidemiological, clinical, imaging and laboratory data, and other data were collected and analyzed. Patients with incomplete clinical data, or those who died of causes unrelated to COVID-19 were excluded from the analysis. Hankou Hospital is a grade III class B hospital designated for COVID-19 treatment, while Huoshenshan Hospital is a hospital specifically built for COVID-19 treatment during the early stage of the epidemic. These patients were divided into two groups for analysis, according to the final outcome: survival group and non-survival group.

### Diagnostic criteria and clinical classification

A patient was diagnosed with COVID-19 based on the following: (1) when the patient had an etiological evidence of SARS-CoV-2 infection, and/or (2) when the patient had an epidemiological history, with any two of the clinical manifestations with or without any of the pulmonary imaging changes described below, according to the 5th or 7th edition of the COVID-19 Diagnosis and Treatment Program of China [9, 10].

An etiological evidence included: (1) positive SARS-CoV-2 nucleic acid detected by real-time fluorescence polymerase chain reaction (PCR) in the respiratory or blood samples; (2) viral gene sequencing highly homologous to the known SARS-CoV-2 in the respiratory or blood samples; or (3) positive serum SARS-CoV-2 specific IgM antibody and IgG antibody.

An epidemiological history included: (1) a history of travel or residence in Wuhan and its surrounding areas, or in other reported epidemic areas within 14 days before the onset of the disease; (2) a history of contact with a confirmed COVID-19 patient within 14 days before the onset of the disease; and (3) a history of contact with a patient from Wuhan and its surrounding areas, or a patient with fever or respiratory symptoms from the reported epidemic community within 14 days before the onset of the disease. The clinical manifestations included fever and/or respiratory symptoms with normal or decreased white blood cell count, or decreased lymphocyte count in the early stage of the disease. The pulmonary imaging changes included multiple small patch shadows and stromal changes in the early stage, which were obvious in the lung periphery, and developed into ground glass shadows, and infiltrating shadows or lung consolidation in severe cases, but rarely with pleural effusion.

The COVID-19 cases were clinically classified as mild, common, severe, or critical type [11]. Mild type was defined when the clinical symptoms were mild, without any imaging changes. Common type was defined when patients had clinical symptoms, such as fever and respiratory infection, with pulmonary imaging changes. Severe type was defined when patients presented with any of the following: (1) a respiratory rate of > 30 rate/min, (2) a pulse oxygen saturation (SpO_2_) of ≤93%, and (3) an arterial oxygen partial pressure to fractional inspired oxygen ratio (the ratio of partial pressure of oxygen to fraction of inspiration O_2_: PaO_2_/FiO_2_) of ≤300 mmHg. Critical type was defined when patients presented with any of the following: (1) respiratory failure requiring mechanical ventilation, (2) shock, and (3) other organ failure requiring intensive care unit (ICU) care. In the present study, patients diagnosed with mild and common types were assigned to the non-severe group, while patients diagnosed with severe and critical types were assigned to the severe group.

### Data collection and statistical analysis

The demographic, epidemiological, clinical, imaging and laboratory data were collected from the electronic medical record system of the hospitals. The categorical variables were expressed in frequency (percentage), and continuous variables were expressed in median (interquartile range) or mean ± standard deviation, when appropriate. The Shapiro–Wilk test was used to judge the distribution of continuous variables before the statistical analysis. The categorical variables were compared using Pearson’s χ^2^-test or Fisher’s exact test, while continuous variables were analyzed by Student *t*-test or Mann Whitney *U*-test, as appropriate. Variables with a *P-*value of ≤0.1 in the univariate analyses were included in the log-binomial model using the backward stepwise method, and merely these independent risk factors were used to establish the nomogram model. The relative risk (RR) and 95% confidence interval (CI) were calculated. The bootstrap self-sampling method was used to internally verify the prediction effect of the model, and the verification result was visually presented in the calibration curve. The discrimination of the nomogram was evaluated using the area under the receiver operating characteristic (ROC) curve (AUC), and a calibration curve was constructed to determine whether the prediction result was consistent with the observation result. All statistical analyses were performed using IBM SPSS statistics 20.0 (SPSS Inc., IL, USA) and R v3.5.2 (R Foundation for Statistical Computing, Vienna, Austria, *http://www.Rproject.org*) through RStudio v1.0.136 (RStudio Inc., MA, USA). The nomogram model and calibrating curve were analyzed using the R software package called rms and cali, respectively. All tests were two sided with an alpha level of 0.05.

## Results

### Characteristics of patients with non-survival and survival COVID-19

A total of 269 COVID-19 patients were hospitalized in Wuhan Hankou Hospital (*n* = 215) and Huoshenshan Hospital (*n* = 54) from January 12, 2020 to March 20, 2020. Among them, 141 (52.4%) patients survived, while 128 (47.6%) patients did not survive. The number of patients (rates) were 125 (58.1%) and 90 (41.9%), respectively, for Hankou Central Hospital, and 16 (29.6%) and 38 (71.4%), respectively, for Huoshenshan Hospital. Seven survived patients in survival group were excluded due to incomplete information. Finally, 262 patients were included in the analysis. Among these patients, 143 patients were male and 119 were female, and their median age was 67 years old (range: 31–96 years old) (Table 1).

**Table 1:** Clinical characteristics and laboratory parameters of patients in the survival and non-survival groups at admission

Clinical features	Total (*n* = 262)	Survival (*n* = 134)	Non-survival (*n* = 128)	*P-*value	Adjusted RR (95%CI)†	*P-*value
Age, years	67 (59, 73.25)	62.5 (51.75, 68.25)	70.5 (65, 78)	< 0.001*	0.905 (0.868, 0.944)	< 0.001*
Gender				0.202		
*Female%*	119 (45.4%)	66 (49.3%)	53 (41.4%)			
*Male%*	143 (54.6%)	68 (50.7%)	75 (58.6%)			
Smoking	14 (5.3%)	3 (2.2%)	11 (8.6%)	0.022*		
Alcohol drinking	7 (2.7%)	2 (1.5%)	5 (3.9%)	0.226		
Illness to length of stay, days	10 (5, 14)	10 (5, 14)	10 (7, 13)	0.962		
Comorbidities						
*Hypertension*	121 (46.2%)	50 (37.3%)	71 (55.5%)	0.003*		
*Diabetes*	56 (21.4%)	24 (17.9%)	32 (25%)	0.162		
*Cardiovascular disease*	45 (17.2%)	12 (9%)	33 (25.8%)	< 0.001*	0.045 (0.010, 0.205)	< 0.001*
*COPD*	7 (2.7%)	1 (0.7%)	6 (4.7%)	0.061		
*Chronic kidney disease*	10 (3.8%)	4 (3%)	6 (4.7%)	0.533		
*Tumor*	11 (4.2%)	3 (2.2%)	8 (6.2%)	0.106		
*Nervous system disease*	15 (6.5%)	5 (3.7%)	12 (9.4%)	0.064		
*Other*	42 (16%)	25 (18.7%)	17 (13.3%)	0.236		
Temperature at admission, ^o^C	38.5 (38, 39)	38.5 (38, 39)	38.6 (38, 39)	0.430		
Duration of fever, day	10 (5, 14)	10 (5, 14)	10 (6.5, 12)	0.867		
Respiratory rate at admission, rate/min	20 (20, 25)	20 (20, 23)	22 (20, 30)	< 0.001*		
SpO_2_ at admission,%	90 (80, 96)	96 (92, 98)	83 (70, 91)	< 0.001*		
Symptoms						
*Fever*	208 (79.4%)	108 (80.6%)	100 (78.1%)	0.621		
*Cough*	190 (72.5%)	98 (73.1%)	92 (71.9%)	0.819		
*Expectoration*	79 (30.2%)	47 (35.1%)	32 (25%)	0.076		
*Pharyngalgia*	10 (3.8%)	7 (5.2%)	3 (2.3%)	0.336		
*Vomit*	10 (3.8%)	4 (3%)	6 (4.7%)	0.533		
*Poor appetite*	115 (43.9%)	65 (48.5%)	50 (39.1%)	0.124		
*Muscular soreness*	24 (9.1%)	12 (8.8%)	12 (9.4%)	0.876		
*Diarrhea*	17 (6.5%)	11 (8.2%)	6 (4.7%)	0.247		
*Headache*	7 (2.7%)	5 (3.7%)	2 (1.6%)	0.448		
*Shortness of breath*	143 (54.6%)	60 (44.8%)	79 (61.7%)	0.001*		
*Weakness*	155 (57%)	82 (61.2%)	73 (57%)	0.493		
Severity of disease at admission				0.191		
*Non-severe*	111 (42.4%)	62 (46.3%)	49 (38.3%)			
*Severe*	151 (57.6%)	72 (53.7%)	79 (61.7%)			
Positive nucleic acid before admission	73 (29%)	36 (26.9%)	37 (31.4%)	0.433		
Positive nucleic acid at admission	99 (37.8%)	49 (49.5%)	50 (39.1%)	0.677		
**Laboratory parameter**						
White blood cell, ×109/L	6.5 (4.5, 10.2)	5.6 (3.9, 7.42)	8.85 (5.67, 12.9)	< 0.001*		
Lymphocyte, ×109/L	0.7 (0.41, 1.0)	0.8 (0.6, 1.15)	0.66 (0.3, 0.8)	< 0.001*		
LYM percentage, %	10 (5.4, 20.525)	16.5 (8.8, 26.2)	6.4 (3.45, 10.25)	< 0.001*	1.125 (1.041, 1.216)	0.0029*
Blood platelet, ×109/L	188 (137, 246)	216.5 (157.5, 265.7)	169 (111, 229)	0.001*	1.008 (1.003, 1.012)	0.001*
Hemoglobin, g/L	126 (117, 137)	126 (117.75, 137.25)	124 (115.5, 137)	0.436		
CRP, mg/L	36.45 (17.54, 101.5)	31.98 (7.52, 37.93)	61.76 (35.52, 148.31)	< 0.001*	0.982 (0.973, 0.991)	< 0.001*
PCT, ng/ml	0.083 (0.05, 0.20)	0.068 (0.039, 0.09)	0.237 (0.09, 0.60)	< 0.001*		
APTT, s	34.5 (30.4, 40.6)	34.3 (31.32, 41.1)	34.85 (29.97, 39.7)	0.603		
Fibrinogen, g/L	3.825 (2.85, 4.67)	3.75 (2.89, 4.73)	3.97 (2.81, 4.67)	0.841		
TT, s	15.3 (14.6, 16.29)	15.3 (14.5, 15.9)	15.3 (14.6, 16.6)	0.287		
PT, s	13.9 (12.85, 15.45)	13.9 (13.0, 15.05)	13.95 (11.95, 15.97)	0.932		
INR	1.14 (1.05, 1.3)	1.12 (1.04, 1.21)	1.2 (1.08, 1.61)	0.002*		
D-dimer, μg/mL	0.75 (0.205, 4.61)	0.31 (0.12, 1.3)	1.22 (0.51, 7.39)	< 0.001*	0.734 (0.617, 0.879)	< 0.001*
CK, U/L	99.5 (59, 201.75)	87 (43, 173)	108.3 (72, 348)	0.065		
CK-MB, U/L	15.7 (11, 27)	14 (9, 19)	19 (13.72, 35.45)	< 0.001*		
a-HBDH, U/L	329.7 (223.1, 461.8)	268.3 (185.85, 364.2)	483.3 (279.75, 609.77)	< 0.001*		
cTnT, ng/mL	0.03 (0.014, 1.395)	0.014 (0.011, 0.022)	0.17 (0.041, 8.13)	< 0.001*		
BNP, pg/mL	199.4 (81.34, 874.3)	118.35 (67.88,280.6)	388.6 (145.2, 2262)	< 0.001*		
TBil, μmol/L	9.7 (7.1, 13.9)	8.7 (6.9, 11.3)	11.1 (7.8, 17.12)	< 0.001*		
DBil, μmol/L	3.9 (2.6, 6.47)	3.3 (2.4, 4.3)	5.05 (3.47, 9.3)	< 0.001*		
IBil, μmol/L	5.5 (3.8, 7.3)	5.3 (3.6, 7.1)	5.85 (3.87, 7.96)	0.186		
ALT, U/L	26.1 (17, 40)	25 (17, 36)	27.1 (18.15, 43.6)	0.151		
AST, U/L	32 (24, 49)	28 (22, 40)	38 (27.4, 61)	< 0.001*		
LDH, U/L	334 (236.9, 476)	269 (199, 382)	422 (294, 584.9)	< 0.001*	0.993 (0.990, 0.997)	< 0.001*
Cr, μmol/l	75 (57.25, 94)	67 (56.4, 84.5)	83 (60, 108)	0.001*		
BUN, mmol/L	5.52 (4.14, 8.29)	4.78 (3.8, 6.51)	7.42 (4.48, 13.24)	< 0.001*		
Alb, g/L	31.7 (29.3, 35.7)	32.8 (30, 36.9)	31 (28.7, 33.95)	0.001*		

The data are expressed in median (interquartile range) or number (%), when appropriate

*Abbreviations*: *COPD* Chronic obstructive pulmonary disease, *CRP* C-reactive protein, *PCT* Procalcitonin, *APTT* Activated partial thromboplastin time, *TT* Thrombin time, *PT* Prothrombin time, *INR* International normalized ratio, *CK* Creatine kinase, *CK-MB* Creatine kinase isoenzyme, *CTnT* Troponin T, *α-HBDH* α-Hydroxybutyrate dehydrogenase, *TBil* Total bilirubin, *IBil* Indirect bilirubin, *DBil* Direct bilirubin, *ALT* Alanine aminotransferase, *AST* Aspartate aminotransferase, *LDH* Lactate dehydrogenase, *Cr* Creatinine, *BUN* Urea nitrogen, *Alb* Albumin, *RR* Relative risk

*P < 0.05 was considered statistically significant

†, Variables with a *P* value of ≤0.1 in the univariate analyses were included in the log-binomial model and statistical data are not shown for variables that were not significant (i.e. *P* > 0.05) in the log-binomial model

There were significant differences in smoking, coronary heart disease (CHD), hypertension, shortness of breath, respiratory rate and SpO_2_ at admission between the survival and non-survival groups (*P* < 0.05) (Table 1). Furthermore, there were significant differences in white blood cell count (WBC), lymphocyte, lymphocyte percentage (Lym%), platelet (PLT), D-dimer, ɑ-hydroxybutyrate dehydrogenase (ɑ-HBDH), international normalized ratio (INR), CK-MB, cTnT, BNP, total bilirubin (TBil), direct bilirubin (DBIL), aspartate aminotransferase (AST), C-reactive protein (CRP), procalcitonin (PCT), lactate dehydrogenase (LDH), creatinine (Cr), urea nitrogen (BUN) and albumin (Alb) between these two groups within 24 h after admission (Table 1).

### Independent risk factors for survival as determined by the multivariate logistic regression analysis

After the univariate analyses, 26 potential risk factors were included in the log-binomial model analysis. Finally, seven variables, including age (RR: 0.905, 95% CI: 0.868–0.944; *P* < 0.001), CHD (RR: 0.045, 95% CI: 0.0097–0.205; *P* < 0.001), Lym% (RR: 1.125, 95% CI: 1.041–1.216; *P* = 0.003), PLT (RR: 1.008, 95% CI: 1.003–1.012; *P* = 0.001), CRP (RR: 0.982, 95% CI: 0.973–0.991; *P* < 0.001), LDH (RR: 0.993, 95% CI: 0.990–0.997; *P* < 0.001), and D-dimer (RR: 0.734, 95% CI: 0.617–0.879; *P* < 0.001), were identified as independent risk factors. These were used to build the nomogram for predicting the survival probability.

### Establishment and verification of the nomogram model

**Fig. 1:**
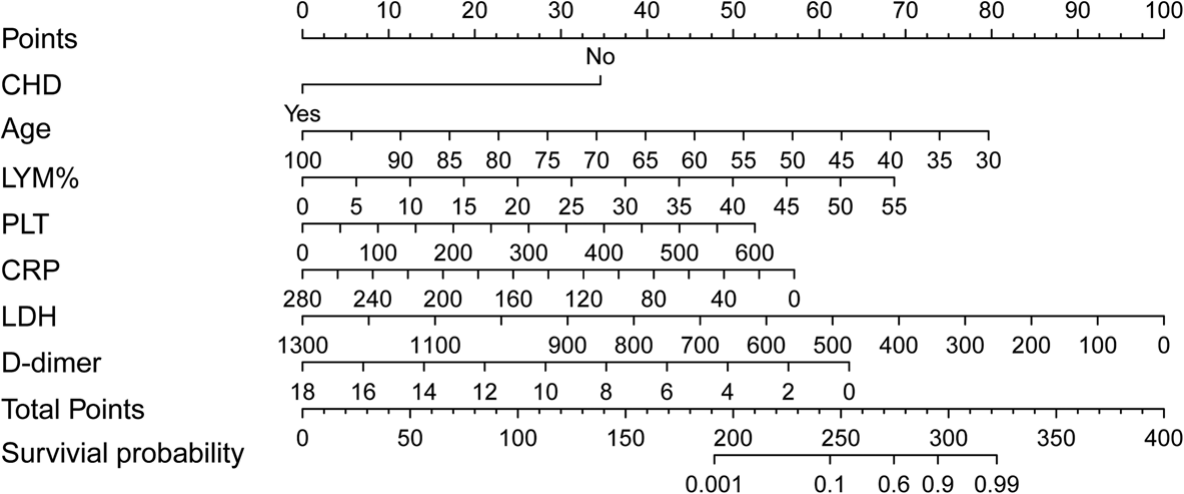
A nomogram for predicting the survival probability of patients with COVID-19. Abbreviations: Age, years; CHD: coronary heart disease; Lym%: lymphocyte percentage; PLT: platelet, × 10^9^/L; CRP: C-reactive protein, mg/L; LDH: lactate dehydrogenase, U/L. Instructions for using the nomogram: (1) Draw a vertical line based on the value of each variable to obtain the corresponding point; (2) Add all seven points to obtain the total point; (3) Draw a vertical line based on the total point to determine the estimated survival probability

The foreign package of the R language was used to read the original data, and the rms package was used to bring age, CHD, Lym%, PLT, CRP, LDH and D-dimer into the functional model, and form the nomogram model for predicting the outcome of COVID-19 patients. The identified seven risk factors were incorporated into the prediction model, and presented as the nomogram in Fig. 1.

**Fig. 2:**
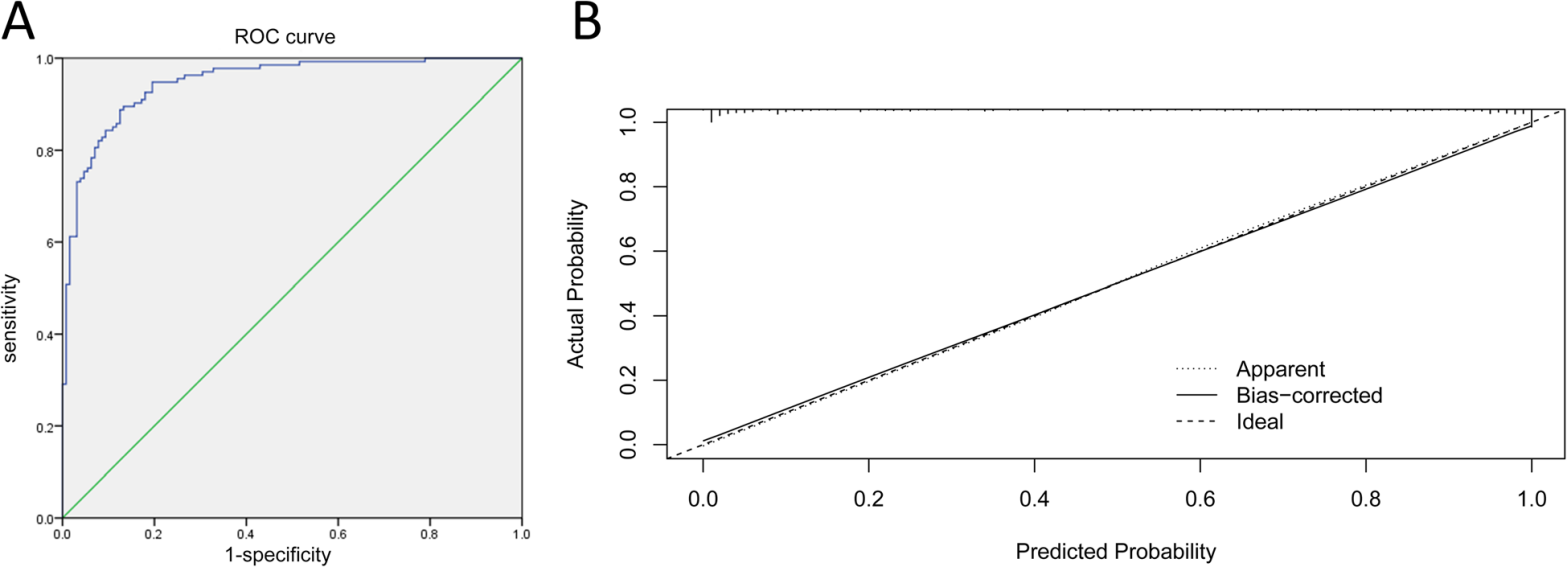
The receiver operating characteristic (ROC) curve (**a**) and calibration curve (**b**) for the established nomogram. OR: odds ratios; AUC: area under the curve; 95% CI: 95% confidence interval. Calibration curve reflects the extent to which the model correctly estimates the absolute probability or agreement between the predicted probability and observed outcomes. The Y-axis represents the actual survival probability. The X-axis represents the predicted survival probability. The black dot at the top represents the prediction probability corresponding to the actual observation, the black dotted line represents the ideal predicted value, and the solid line represents the actual predicted value

The ROC curve was generated for the nomogram, with an AUC of 0.948 (95% CI: 0.923–0.973, Fig. 2a). The internal verification using the bootstrap method was visually presented in the calibration curve (Fig. 2b). This revealed that the prediction probability of the nomogram was consistent with the actual situation. The calibration curve was slightly nonlinear. Furthermore, the consistency was better in the range of predicting the survival probability of patients < 50%, while the inconsistency slightly increased in the range of predicting the survival probability of patients ≥50%.

## Discussion

In the present study, 262 patients with COVID-19, who were admitted in two designated hospitals, and either survived or died during the hospitalization, were analyzed. The multivariate logistic regression analysis identified age, CHD, and the five laboratory variables, which included Lym%, PLT, CRP, LDH and D-dimer, as independent factors for the survival of COVID-19 patients. Based on this, a nomogram for predicting in-hospital survival was established, with excellent performance.

Advanced age has been reported as an important independent predictor of SARS and MERS mortality [12]. Several studies have revealed that elderly patients and patients with underlying comorbidities tend to have poor outcomes [13–16]. The patients infected with SARS-CoV-2 were analyzed, and it was found that there was a significant difference in age between the survival and non-survival groups. Furthermore, the multivariate analysis also revealed that age was an independent factor that affected the prognosis of COVID-19 patients. In the nomogram established by our team, it was found that the survival rate of COVID-19 patients decreased with the increase in age. According to the present analysis of the clinical data, the proportions of hypertension and CHD were significantly higher in the non-survival group, when compared to the survival group, and CHD was an independent risk factor for the prognosis of COVID-19 patients. This suggests that patients with hypertension and CHD are susceptible to SARS-CoV-2 infection, and have a high mortality rate. Although the underlying mechanism remains unclear, this may have been contributed by the following causes. First, Angiotensin converting enzyme 2 (ACE2) is widely expressed in myocardial cells, myocardial fibroblasts and coronary endothelial cells. It has been reported that the Spike (S) protein of SARS-CoV-2 binds with high affinity to human ACE2, and before SARS-CoV-2’s entry into the cells [17–19], the S protein is subjected to a priming process via serine protease TMPRSS2 in order to permit the attachment of viral particles to ACE2 and thus on cell surface [20, 21]. Therefore, SARS-CoV-2 can directly attack cardiac muscle cells through this pathway [22–24]. This entry mechanism is confirmed by the fact that TMPRSS2 inhibition or TMPRSS2-KO mice show both decreased, though not abolished, S protein priming, and reduced viral entry, spread, as well as, inflammatory chemokine and cytokine release [20]. Second, there may be an imbalance between Th1 and Th2 responses in COVID-19 patients, and the cytokine storm triggered by this imbalance may be another mechanism of myocardial injury [25–27].

Interestingly, it was found PLT and D-dimer were independent risk factors for predicting the outcome of COVID-19 patients. Thrombocytopenia has been reported to be present in up to 55% of patients with SARS, and has been identified as an important risk factor for mortality [28]. In a study conducted by Zou et al., merely two variables (i.e. platelet count and hypoxemia) were used to establish a SARS prognostic model, and this was used to predict the survival rate, with an accuracy of 96.2% [5]. It has been demonstrated that the pathological features of COVID-19 are similar to those of SARS and MERS [29]. In the early stage of infection, patients present with inflammation, edema, protein exudation, focal hyperplasia of alveolar epithelial cells and patchy inflammatory infiltration, as well as multi-nucleated giant cells in the lung. In the late stage, in addition to hemorrhage and some areas of interstitial fibrosis, diffuse alveolar damage was also observed [30]. Furthermore, fibrous clots and gelatinous mucus in the small airway and diffuse intravascular coagulation were observed [31]. In a previous study, it was found that lung injury was the main cause, followed by heart, liver and kidney injuries, and coagulation system abnormality [32]. In the present study, there were abnormal coagulation functions in both the survival and non-survival groups, which mainly manifested with the increase in D-dimer. At the same time, it was found that there was a significant increase in fibrin degradation products and thrombocytopenia, which was consistent with the presence of hyper-fibrinolysis, in patients with severe COVID-19 [33]. Therefore, it is not difficult to understand that platelets and D-dimers are independent risk factors that affect the outcome of patients. Noteworthy, patients with severe SARS-CoV-2 infection often possess coagulation dysfunction at admission [34]. A recent study by Zhang et al demonstrated that INR was a prognostic factor for clinical outcomes in patients with severe COVID-19 [34]. In the present study, we observed that there was also significant in the INR between survival and non-survival group in univariate analysis. However, it was not an independent risk factor for the survival of COVID-19 patients in multivariate analysis. Thus, further investigation is required to reveal the association between coagulation disorder and adverse clinical outcome in severe COVID-19 patients.

The present study revealed that Lym% and CRP were also independent risk factors that affected the prognosis of patients. The decrease in Lym% indicates that the immune system of a patient with SARS-CoV-2 is more likely to be suppressed, and that the host loses the immune function to fight against the pathogen, leading to the persistence of the infection and deterioration of COVID-19. In addition, the elevated levels of CRP observed in the present study, as well as in previous studies [32, 35], also suggest that there is a persistent inflammatory response in COVID-19 patients. Finally, it was found that LDH was another independent risk factor to predict the outcome of COVID-19 patients. It has been shown that COVID-19 patients first present with lung injury, which subsequently leads to hypoxemia and multiple organ damage [36]. Consequently, LDH in cells is released, resulting in increased LDH levels. Indeed, LDH was used to predict the severity of tissue damage in the early stage of diseases as an auxiliary marker, and for the early identification of cases at high risk of progression to severe COVID-19 [37].

The nomogram, as a visual form of predictive models, has been used in the diagnosis, treatment and prognosis of various diseases [6–8, 38]. It is visual and intuitive, and does not need to substitute the numbers into the equation calculation. Furthermore, the user only needs to draw one or more lines to quickly and reliably obtain the prediction. In the present study, a nomogram was established, which can be used to accurately predict the prognosis of COVID-19 patients. The calibration curve revealed the accuracy of the nomogramic model for predicting the prognosis of COVID-19 patients (“dominant state”) and the bootstrap model (“bias correction state”), which can explain the relationship between the prediction probability and actual observation probability in the original data set.

There were a few limitations in the present study. First, this was a retrospective study with relatively a small sample size. Second, the nomogram in the present study was not externally verified through another cohort of COVID-19 patients. Thus, the predictive performance remains to be further confirmed. Third, patients included in the present study were all over 18 years old. Thus, the nomogram is not suitable for children and pregnant women.

## Conclusions

A nomogram based on age, CHD, Lym%, PLT, CRP, LDH and D-dimer was established to accurately predict the prognosis of COVID-19 patients. In clinical practice, the survival probability of COVID-19 patients can be obtained by simple calculation. This can be used as an alerting tool for clinicians to take early intervention measures, when necessary.

## Data Availability

The datasets used and analyzed during the current study are available from the corresponding author on reasonable request.

## Abbreviations

ARDS: Acute respiratory distress syndrome
Alb: Albumin
ACE2: Angiotensin converting enzyme 2
AST: Aspartate aminotransferase
ɑ-HBDH: ɑ-hydroxybutyrate dehydrogenase
BUN: Urea nitrogen
COVID-19: Coronavirus disease 2019
CHD: Coronary heart disease
CRP: C-reactive protein
Cr: Creatinine
DBIL: Direct bilirubin
ICU: Intensive care unit
INR: International normalized ratio
LDH: Lactate dehydrogenase
Lym%: Lymphocyte percentage
PCR: Polymerase chain reaction
PLT: Platelet
PCT: Procalcitonin
PaO_2_/FiO_2_: The ratio of partial pressure of oxygen to fraction of inspiration O_2_
WBC: White blood cell count
RR: Relative risk
ROC: Receiver operating characteristic curve
SARS-CoV-2: Severe acute respiratory syndrome coronavirus 2
SpO_2_: Pulse oxygen saturation
S protein: Spike protein
TBil: Total bilirubin
